# The Poet’s Gift of Consolation to Sorrowing Mothers

**Published:** 1874-02

**Authors:** 


THE POET'S GIFT OF CONSOLATION TO SORROWING MOTHERS, is the title of a beautifully bound volume of poems published by A. S. Barnes & Co., New York. This beautiful book is filled with poems taken from Mrs. Browning, Longfellow, Lowell, Gerald Massey, Timrod, Dean, Trench, and many others, making as rich a collection and as charming a grouping of poetry as "Sorrowing Mothers" could wish. Price $1.50.

